# An Evolution Based on Various Energy Strategies

**DOI:** 10.3390/e23030317

**Published:** 2021-03-08

**Authors:** Alexander O. Gusev, Leonid M. Martyushev

**Affiliations:** 1 Technical Physics Department, Ural Federal University, 19 Mira St., 620002 Ekaterinburg, Russia; xpbim3@gmail.com; 2 Institute of Industrial Ecology, Russian Academy of Sciences, 20 S. Kovalevskaya St., 620219 Ekaterinburg, Russia

**Keywords:** Darwin evolution, maximum entropy production principle, Lotka and Odum principles

## Abstract

The simplest model of the evolution of agents with different energy strategies is considered. The model is based on the most general thermodynamic ideas and includes the procedures for selection, inheritance, and variability. The problem of finding a universal strategy (principle) as a selection of possible competing strategies is solved. It is shown that when there is non-equilibrium between the medium and agents, a direction in the evolution of agents arises, but at the same time, depending on the conditions of the evolution, different strategies can be successful. However, for this case, the simulation results reveal that in the presence of significant competition of agents, the strategy that has the maximum total energy dissipation of agents arising as a result of evolution turns out to be successful. Thus, it is not the specific strategy that is universal, but the maximization of dissipation. This result discovers an interesting connection between the basic principles of Darwin–Wallace evolution and the maximum entropy production principle.

## 1. Introduction

The whole world around us is constantly evolving. We are witnesses and participants in the evolution of both the inanimate and the living world. However, what are the principles of this evolution? This is the most important issue that humanity has long been interested in. We can assume that the whole world around us does not have any permanent principles for its development (or, which is practically the same, the principles change randomly over time). Even with such a simple answer, however, some dissatisfaction still remains. This feeling is due to the fact that the observed world appears to us to be developing unidirectionally. Physicists have expressed this property using the second law of thermodynamics, and biologists refer to the progressiveness of evolution, i.e., the gradual appearance of more complex living systems over time. Based on these observations, some time ago, it was falsely considered that the world of physics tends to chaos, while the world of biology, on the contrary, develops from chaos to order. However, nowadays, this misunderstanding has been completely overcome. It was associated with the erroneous interpretation of thermodynamic entropy as a measure of chaos [1,2]. Now we can say that both the world of the inanimate and the world of the living are developing in one direction [2,3]. This is largely attributed to the maximum entropy production principle (MEPP) [2,3,4,5,6,7]. According to its generalized formulation, at each level of description, with preset external constraints, a local relationship between the cause and the response of a non-equilibrium system is established in order to maximize the specific entropy production. In short, in its development, nature prefers systems that produce more and more entropy per unit.

A discussion of the relationship between the MEPP and the principles that have emerged in evolutionary biology specifically to explain the direction of the evolution of biological species can be found in [2,3,4,6,8,9]. The most famous is the principle of A. Lotka—introduced in 1922—that the evolution proceeds in such a direction as to make the total energy flux through the system a maximum compatible with the constraints [10]. Further development of this principle is the principle suggested by H. Odum [11]: In competition with the other systems, the one that uses more energy and consumes it in the most efficient way survives. Both Lotka and Odum refer to energy as free energy [9]. MEPP is related to the above principles with the fact that the consumption of more energy will inevitably lead to an increase in its dissipation in the form of heat, i.e., entropy production (the change in the energy conversion efficiency (ECE) of systems is extremely limited due to thermodynamic constraints, similarly to the Carnot cycle). At the same time, it is obvious that the evolution of systems according to the MEPP and the principles of Lotka and Odum will be different. This issue has been discussed in the literature [4,8,9] repeatedly, but only speculatively, without calculations based on specific models.

The MEPP and Lotka’s and Odums’s principles (strategies) of development are usually considered as a certain law of nature, which we have discovered (suggested) on the basis of observations and analysis of the surrounding world. However, the question arises: Why does exactly one of these principles exist in nature, and not another one? Will this principle change depending on time or other reasons (for example, during the origin and evolution of life on other planets in substantially different conditions compared to the Earth)? This question is usually avoided in the scientific literature by referring to it as metaphysical. In this paper, for the first time, an approach is proposed to address this issue. This approach is based on a very productive idea expressed by Ch. Darwin and A. Wallace: the selection (survival) of the fittest. This idea is proposed to be applied here as follows. Let there be some set of simultaneously existing agents (organisms) with different strategies. These agents are located in a certain nutrient medium, where they compete, grow, and multiply in accordance with their strategies (principles). There are several possible long-term results of natural selection/competition in such a development. First, none of the strategies will be viable. Second, there will be several such viable strategies. These results will indicate either the absence of a single universal principle of development or some methodological error in our attempt to obtain a principle based on the previously extremely successful Darwin–Wallace theory of selection. However, a very interesting third option is possible: During the selection process, the winners will always be those systems that follow some specific strategy. Naturally, within the approach accepted in the work, this strategy can be considered the principle of evolution. It is especially important that, unlike previous approaches, this principle is not postulated by us a priori for modeling (analyzing) the evolution, but is selected in the course of modeling the evolution itself.

The aim of this paper is an attempt to obtain the principle of evolution based on selection of agents with various strategies. The work will use the simplest thermodynamic model based on the most general laws. Using this model, the case of evolution in a medium with limited resources will be considered in this work. This case is relevant in the evolution of various systems, including a number of biological ones. It is important to note that any model (by its definition) is extremely far from its real prototype (this especially applies to biological systems). Hence, the proposed model can only describe many biological systems very roughly.

## 2. The Model

Let thermal agents with a number *N* evolve in a nutrient medium that includes some nutrient component with an initial number $M_{0}$. Each component has an energy value *L*. Each agent consumes *m* components per unit of time (one iteration), while the energy conversion efficiency (ECE) of one component is η. To extract the component from the medium, energy is needed, which depends on the concentration of the component. We define this dependence as $lM_{0}/M$, where *l* is the energy to extract one component from the medium when its current amount M coincides with $M_{0}$. Thus, per unit of time:

- The energy accumulated by the agent is
  (1) $$\eta mL-mlM_{0}/M.$$
- The total energy flux through the agent is Lm.
- The energy dissipated by the agent into the medium is L(1−η)m.

During its development, as soon as the agent accumulates 2mL energy, it transforms into two agents, each of which has zero initial accumulated energy (the “parent’s” accumulated energy is used on the development of the parent agent, as well as on its transformation and the creation of the “newborn” agents). For these “newborn” agents, the parameters η,m are distributed near the “parental” values based on one of four strategies:

- Two pairs of values maximizing Lm are selected from 100 randomly generated value pairs {η,m} (L-strategy);
- Two pairs of values maximizing Lηm are selected from 100 randomly generated value pairs {η,m} (O-strategy);
- Two pairs of values maximizing L(1−η)m are selected from 100 randomly generated value pairs {η,m} (M-strategy);
- Two pairs of values {η,m} are randomly generated (R-strategy).

It is important to note that the L- and O-strategies of this model are directly related to Lotka’s and Odum’s strategies, respectively. However, the connection between the M-strategy and the maximum entropy production principle is very indirect, since two “newborn” agent systems are outside the scope of the MEPP’s applicability [5,7]. Indeed, the emerging agents form a compound system, not a complex system. This is due to the fact that, in the model, each of the two newborn agents is independent of the other at the moment of their birth; as a consequence, their dissipations do not depend on each other at times close to the moment of birth (mutual influence is negligible). Note that when considering the total dissipation in a system of a very large number of agents at significant time intervals (for times significantly longer than the time between transformations), the mutual influence of agents can no longer be neglected, and they no longer convert energy independently of each other. The so-called emergent effect occurs and, in this case, the condition for complexity, which is required for the MEPP, is satisfied for the system [5,7]. It should also be noted that all agents and the medium considered in the work have the same temperature (in this case, the behavior of energy dissipation and entropy production is similar).

For pairs {η,m}, which characterize “newborn” agents, the random generation of both η and *m* occurs according to the normal law. The modes of this distribution are the parent values $\eta^{*}$ and $m^{*}$. The standard deviation $\Delta m_{0}$ for *m* is assumed to be $D_{m}m^{*}$ and the standard deviation $\Delta \eta_{0}$ for η is assumed to be $D_{\eta }\left(1-\eta^{*}\right)\eta^{*}$ (such a complication was taken in order to prevent the appearance of values η outside the range from 0 to 1). These rules of random generation for η and *m* are applied to simulate such important properties of Darwinian evolution as variability and inheritance. The numerical values of parameters $D_{m}$, $D_{\eta }$ were in the range 0.01–0.1. These values were chosen empirically based on test runs of the model. Upper and lower limits were chosen on the assumption that, during the evolution, changes in η and *m*, on the one hand, were significant, and on the other, the number of emerging agents of each strategy was about a thousand or more. Parameters $D_{m}$, $D_{\eta }$ can be interpreted in two ways. On the one hand, they can only characterize the magnitude of external random impacts on the developing system. On the other hand, for the L-, O-, and M-strategies, they can be considered as the degree of “aggressiveness” of the medium in relation to the agent (the higher $D_{m}$, $D_{\eta }$, the more dissimilar the medium is for the agent and the more significantly the agent, according to its strategy, directionally modifies (adapts) to this medium). Thus, for the L-, O-, and M-strategies, $D_{m}$, $D_{\eta }$ can be considered as a degree of non-equilibrium between the agent and the medium, and so, they are the most important control parameters of the present model.

In addition to the parameters $D_{m},D_{\eta }$ the model has a parameter L/l, which characterizes the energy availability for the agent. The lower limit of this parameter, at which the development of an agent is possible (an increase in its energy and transformation), is determined according to Equation (1) by the condition $\eta L/l>M_{0}/M$. If this inequality is not met for the agent, then the agent goes into hibernation. It is important to note that for lower values of L/l, the interaction between agents is more significant than for higher ones, since the influence of the second term in Equation (1) increases.

The success of the strategy of the evolving system will be defined by two quantities: a number of agents of each strategy $\left(N_{S}\right)$ and a change in the value of the spread (variability) of η and *m*. This change we define as $R=\left(\Delta \eta \Delta m\right)/\left(\Delta \eta_{0}\Delta m_{0}\right)$, where Δη and Δm are the interquartile ranges of η and *m* that occur as a result of evolution of agents. These parameters are directly related to the sustainability of the strategy’s development. Higher values of $N_{S}$ and *R* enable agents of this strategy to be more likely to be successful, particularly, with possible changes in the environment (e.g., if variability is very small, then any changes in the medium are catastrophic for agents of a given population; a population’s variety is an important characteristic of its ability to survive). The presence of two parameters leads to the complexity and cumbersomeness of the analysis of the simulation results. Hence, they were grouped into one parameter for convenience: $S=(N_{S}/N_{m}+R/R_{m})/2$, where $N_{m},R_{m}$ are the maximum values attainable by one of the four strategies under the specified initial conditions. In the selected integral parameter *S*, the equal significance of both terms is considered; its maximum value assumes the greatest success of the strategy.

At the initial moment, the medium contains one agent from each of the four (L-, M, O-, and R-) strategies; the values $\eta_{0},m_{0}$ of these agents are the same. In the process of evolution, *N* increases, *M* decreases, and the calculation stops as soon as the agents of each strategy stop multiplying. The initial value of $M_{0}$ is always assumed to be constant and equal to $5\times 10^{5}$ (with $M_{0}/m=5\times 10^{4}-5\times 10^{5}$). This value allowed us to provide a sufficiently large number of simulation cycles and a large number of agents.

It is important to note that in the considered one-dimensional (in space) model, agents do not interact directly with each other. They interact indirectly by consuming a common nutrient component, M. This indirect interaction is explicitly considered in Equation (1).

## 3. Results and Analysis

**Figure 1:**
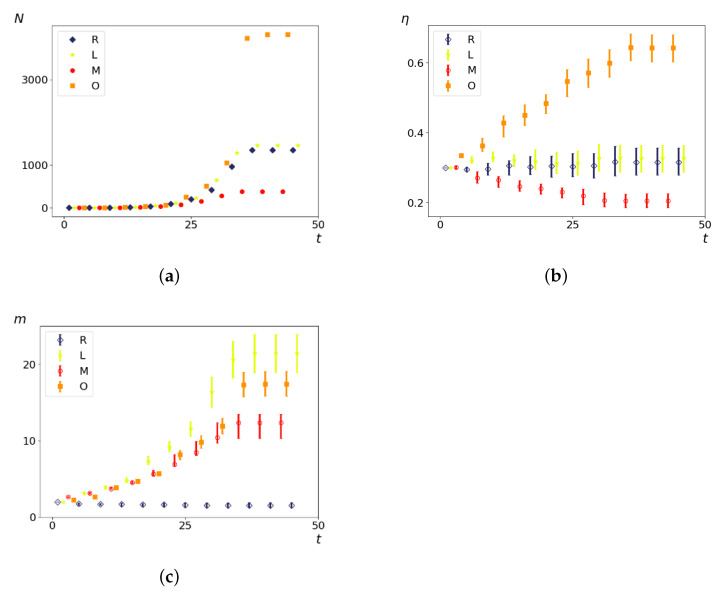
The number of agents *N* (**a**), median energy conversion efficiency (ECE) value η (**b**), and median consumed component *m* (**c**) versus time *t* for four strategies. Interquartile ranges are shown in (**b**,**c**). $\eta_{0}=0.3,m_{0}=2$, $D_{m}=D_{\eta }=0.1,L/l=10.$

All calculations were carried out for agents with initial parameters $\eta_{0}$, $m_{0}$ located within the intervals (0, 1) and [0–10], respectively. An example of the dependencies of the number of agents, their ECE values, and the consumed component on time for different strategies is demonstrated in Figure 1. It can be seen that the parameters that characterize agents change significantly differently for various strategies over time.

**Figure 2:**
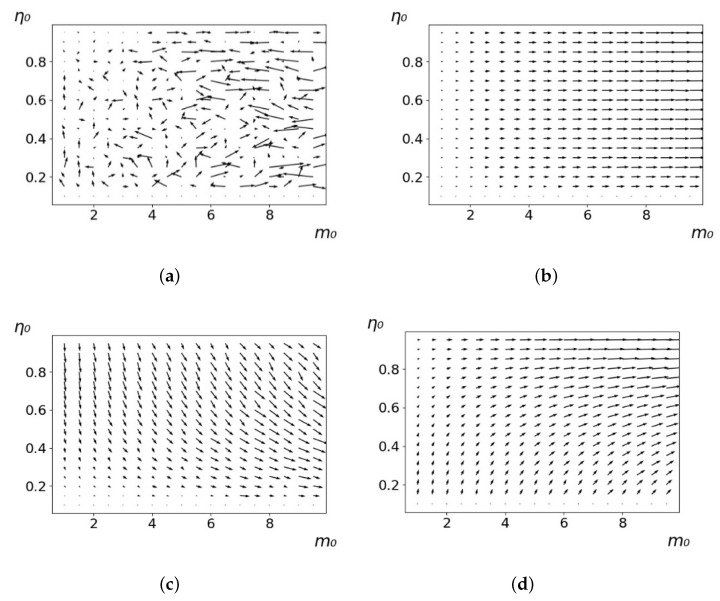
Evolution maps of the R-strategy (**a**), L-strategy (**b**), M-strategy (**c**), and O-strategy (**d**). The arrows show the direction of change in the median values of *m* and η for the corresponding strategies. The initial values of agents are $m_{0}$ and $\eta_{0}$. $D_{m}=0.1,D_{\eta }=0.01,L/l=10.$

As a result of an evolution that ends when the initial resource $M_{0}$ is almost completely exhausted, the parameters of the agents of each strategy change on average. In this case, for the L-, M-, and O-strategies, the change is directed, and for the R-strategy, it is not directed. An example is demonstrated in Figure 2. It shows that for the L-strategy, agents evolve, on average, with increasing *m*; for the M-strategy, in the process of evolution, *m* increases and η decreases, and for the O-strategy, both *m* and η increase. This result is obvious from the nature of the strategies. From the presented maps, it is clear that with multiple repetitions of the experiment (i.e., with multiple additions of the resource $M_{0}$ after its exhaustion), the parameters of the L-strategy agents evolve on average, tending to the maximum possible *m*; for the M-strategy, in the process of evolution, *m* is maximized and η is minimized, and for the O-strategy, both *m* and η are maximized.

**Figure 3:**
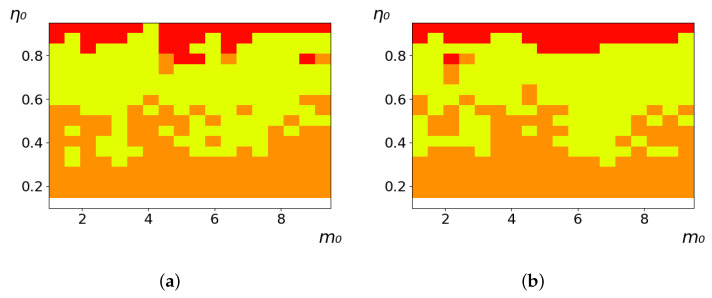

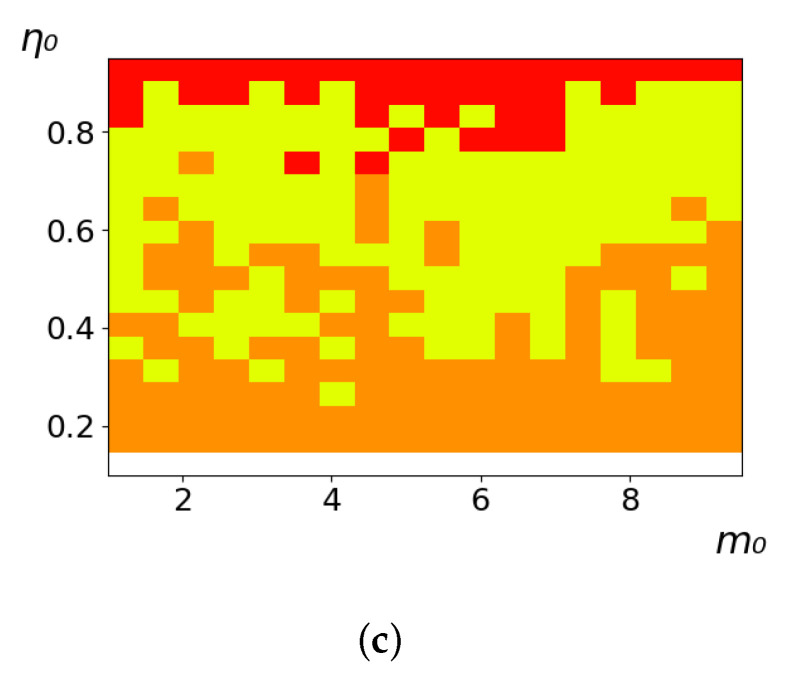
Maps of the leadership of strategies based on the calculation of *S*, Three calculation results (**a**–**c**) are demonstrated. These calculations correspond to three independent simulations with the same parameters and show that due to the stochasticity inherent in the model, the maps change quantitatively, but not qualitatively. The color denotes the winner for the corresponding initial values of *m*_0_ and *η*_0_. Orange, yellow, and red colors correspond to the O-, L-, and M-strategies, respectively. *D_m_* = *D_η_* = 0.1, L/l=10.

As follows from the calculations, the success of a particular strategy depends significantly on the initial values of an agent. This is most clearly demonstrated using maps, where a certain color denotes the area of initial values of $m_{0}$ and $\eta_{0}$, where a particular strategy has the highest value *S* (i.e., it is the most successful). Despite the fact that the presented simulation is stochastic in nature, these maps practically do not qualitatively change with multiple repetitions of calculations (for example, see Figure 3). The maps shown in this figure are quite interesting. Three areas can be distinguished: For small values of $\eta_{0}$, the winner is the O-strategy; for medium values of $\eta_{0}$, the winner is the L-strategy; and finally, for large values of $\eta_{0}$, the winner is the M-strategy. Comparing these results with the vector maps in Figure 2, it can be concluded that the agents of winning strategies from the lower and upper regions of the map in the process of evolution tend to get into the middle zone. As a result, with multiple additions of the resource $M_{0}$, agents will gradually increase the value of *m*, keeping the average values of η. This evolution is extremely successful, since the parameters of the L-strategy agents change towards the values, due to which the L-strategy still remains the leader. This is an example of a definitely successful directed evolution of the agents with the L-strategy. It is easy to imagine numerous other cases—for example, a strategy striving for an area where it is not capable of being a leader (this is an example of a directed but generally disastrous evolutionary strategy).

The results of calculating the maps of successful strategies depending on the initial values of agents $\eta_{0}$, $m_{0}$ are shown in Table 1 and Table 2, respectively, for L/l=10 and L/l=100. These values correspond to two cases: poorly and easily available energy for an agent. It can be seen that the success of the strategy is not strongly dependent on the parameter L/l. Analysis of the results presented in Table 1 and Table 2 and Figure 2, as well as other modeling data, leads to the following conclusions:

1. The success of a strategy (formalized by the parameter *S*) for small initial values of ECE is, in most cases, determined by the number of agents in this strategy. For medium and large initial values of ECE, the success of the strategy is determined mainly by the parameter *R*.
2. Directed and successful strategies (in terms of S) in the case of multiple repetitions of the initial conditions (renewal of the initial resource $M_{0}$) are cases related to rather large values of $D_{\eta }$ and $D_{m}$ (first {1; 0.1} and second {0.1; 0.1} rows in Table 1 and Table 2). Here, the agents that belong to the L-strategy can be considered the winners (see the discussion of Figure 3 above). Such cases of relatively large $D_{\eta }$ and $D_{m}$, as mentioned above, characterize a relatively large degree of non-equilibrium of the medium with respect to the agents located in it. The behavior of agents corresponding to $D_{\eta }=0.01$ and $D_{m}=0.1$ (third row in Table 1 and Table 2) is self-defeating in general, when the initial resource is repeatedly renewed. This is due to the fact that the areas where the winners are the L-, M-, and O-strategies are randomly located on the map in very small ranges. As a result, with a large number of repetitions of the initial conditions, a continuous random change of the successful strategy occurs, which leads to a decrease in the number of agents. For relatively small non-equilibria, {0.1; 0.01} and {0.01; 0.001} (see the last two rows in Table 1 and Table 2), the behavior of agents is not directional. It is chaotic, since the attractor area here is the dominant R-strategy, which, as can be seen from Figure 2a, is characterized by a random change in *m* and η, both in direction and in modulus.
3. For directed and successful strategies (first two rows in Table 1), in the case of L/l=10, the map of the leadership of strategies based on the value of *S* coincides rather well with the map of strategies that, according to the results of evolution, have the maximum total dissipation (comparing the first and last columns in the specified rows). For directed strategies, in the case of L/l=100, this match is worse (see first and last columns at first two rows in Table 2). As noted above, the mutual influence between agents is more significant for the case L/l=10 and in this case for large time of evolution a condition for MEPP applicability is satisfied. Thus, in this simulation, it is shown that in the case of sufficient non-equilibrium and interactions between agents, the most successful strategies, which have been selected in the course of evolution, have the maximum energy dissipation (entropy production) as well as a directed change in the characteristics of agents. This is a very interesting, important and unexpected result. Note (see Table 1 and Table 2), that the mentioned coincidence with the *S* map, is presented only by the maximum dissipation map. Other energy characteristics, such as ηm and *m* do not demonstrate such a coincidence (comparing maps of the first column with maps in the second and third columns).
4. The calculation results (Table 1 and Table 2) clearly demonstrate that a strategy that tends to maximize any energy characteristic from a “parent” to its direct descendants does not often reach the maximum of this characteristic as a result, which is calculated in total over a sufficiently long time interval for all agents of the selected strategy. This can be seen from the presented maps, since the maximum total dissipation mostly does not appear for the M-strategy agents (for example, for $\left\{D_{\eta };D_{m}\right\}$={1;0.1}, the map presented in the last column of Table 1 and Table 2 is almost all yellow, not red), the maximum energy consumed does not appear for the L-strategy agents (for example, for $\left\{D_{\eta };D_{m}\right\}$={1;0.1}, the map presented in the third column of Table 1 and Table 2 is mostly orange rather than yellow), etc. This is due to the mutual influence of agents on each other, which increases itself with a longer amount of time of the considered evolution compared to the average time between transformations of the “parent” agent.

## 4. Conclusions

The paper presents the simplest one-dimensional model, which is based on the most general energy rules and parameters. This model allows us to abstract from the extremely diverse and specific competition in space and focus exclusively on the “energy” competition in time, which is quite universal in nature. The most important and generally accepted characteristics of evolution, such as selection, inheritance, and variability, were included in this model. On the basis of this model, a simulation of the evolution of agents with different strategies was performed. It was assumed that agents compete with each other in a medium with limited resources.

Despite the simplicity of the model, the results turned out to be rather non-obvious and non-linear. It was shown that, when there is a non-equilibrium between the medium and the agents, a direction in the evolution of the agents appears, but there is no major strategy. For example, for small initial values of agents’ ECE, the most successful strategy is Odum’s strategy, and for average initial values of ECE, it is Lotka’s strategy. However, the simulation results demonstrate that, in the presence of rivalry and selection during evolution, the strategy that has the maximum total dissipation of agents turns out to be successful. Thus, it is not a specific strategy—for example, Lotka’s or Odum’s—that is universal in nature, but a tendency to maximize dissipation (entropy production). This result specifies a strong connection between the principles of evolution proposed by Darwin–Wallace and the MEPP for non-equilibrium competitively evolving systems. This connection is an additional argument regarding the applicability of the MEPP for the analysis of biological evolution, which is sometimes questionable [12].

In this work, an important and new method was proposed to validate a principle, and it turned out to be a consequence of the selection of possible competing strategies in nature. This approach definitely requires further analysis and development. One of the possible directions is the discussion and verification of the results obtained in this study using the analytical formalism based on Price’s equation (which is important in mathematical evolutionary biology) [13,14,15,16]. It is interesting to note that earlier, in [13,14], some similarities between this mathematical formalism and the MEPP were pointed out.

It is also necessary to point out here that the model considered in this work has such general properties that its application should not be associated only with biology. Agents can include objects related to various fields of science, from technology and economics (e.g., competing firms, technologies) to the humanities (e.g., development and evolution of scientific theories).

## Figures and Tables

**Table 1:** Maps of the leadership of strategies based on the value of *S*, as well as the total useful, consumed, and dissipated energy. Summation is carried out for all agents of each strategy during the entire simulation time. Orange, yellow, red, and blue colors correspond to the O-, L-, M-, and R-strategies, respectively. L/l=10 (L=0.5).

{$D_{\eta }$; $D_{m}$}	Strategies with Maximum *S*	Strategies with Maximum Total Useful Energy, Σ(ηmL)	Strategies with Maximum Total Consumed Energy, Σ(mL)	Strategies with the Maximum Total Dissipation, Σ(1−η)mL
{1; 0.1}	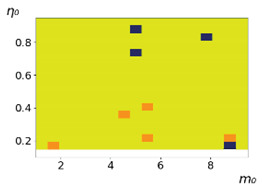	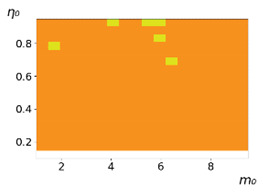	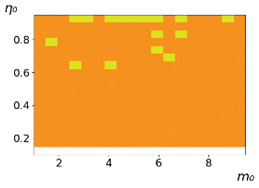	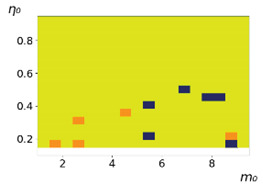
{0.1; 0.1}	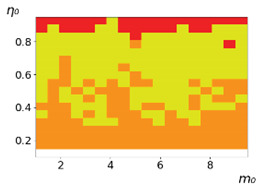	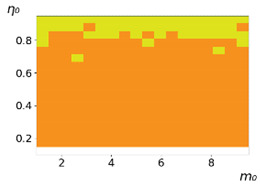	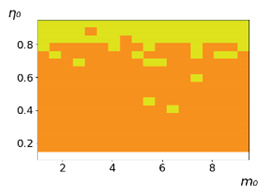	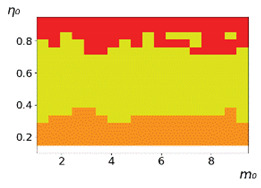
{0.01; 0.1}	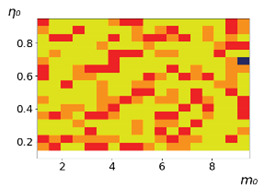	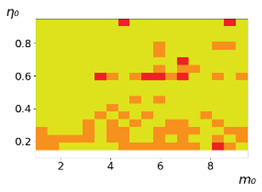	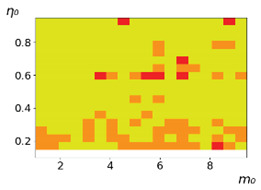	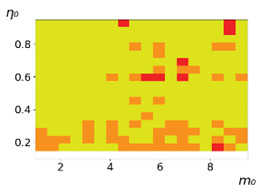
{0.1; 0.01}	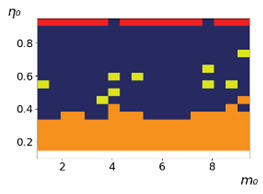	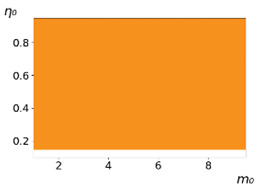	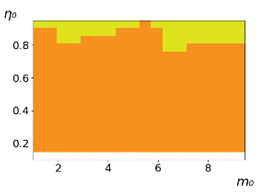	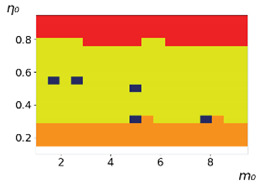
{0.01; 0.001}	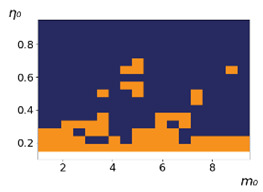	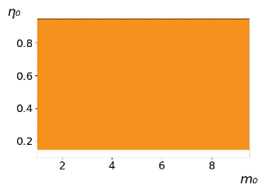	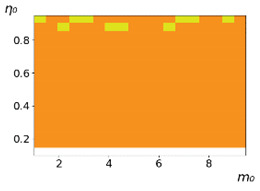	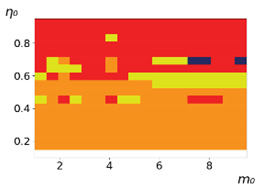

**Table 2:** Maps of the leadership of strategies based on the value of *S*, as well as total useful, consumed and dissipated energy. Summation is carried out for all agents of each strategy during the entire simulation time. Orange, yellow, red, and blue colors correspond to the O-, L-, M- and R-strategies, respectively. L/l=100 (L=0.5).

{$D_{\eta }$; $D_{m}$}	Strategies with Maximum *S*	Strategies with Maximum Total Useful Energy, Σ(ηmL)	Strategies with Maximum Total Consumed Energy, Σ(mL)	Strategies with the Maximum Total Dissipation, Σ(1−η)mL
{1; 0.1}	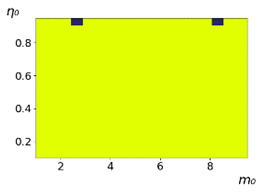	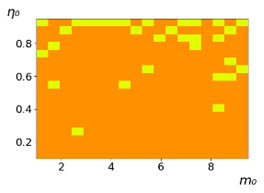	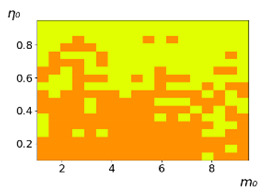	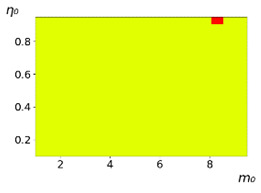
{0.1; 0.1}	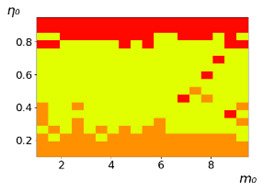	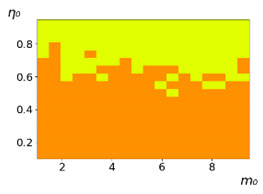	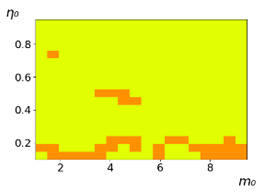	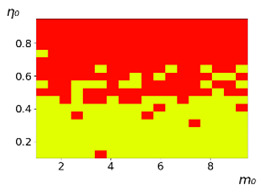
{0.01; 0.1}	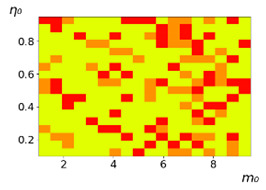	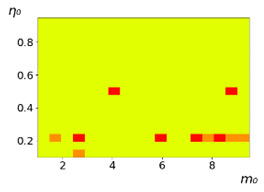	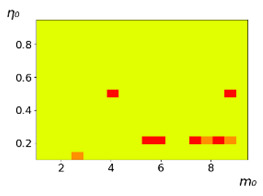	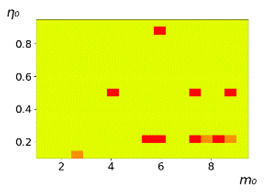
{0.1; 0.01}	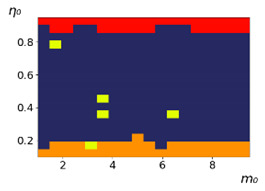	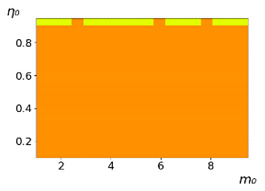	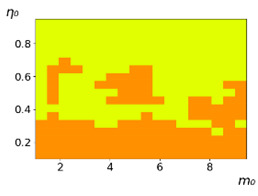	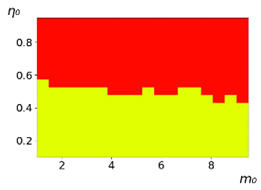
{0.01; 0.001}	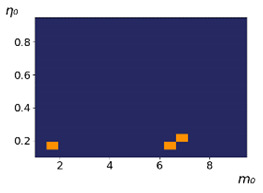	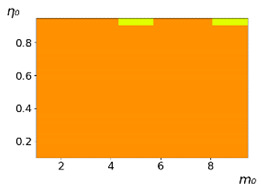	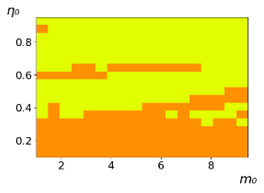	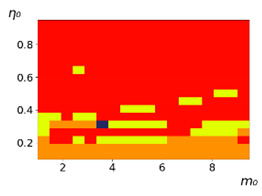

## Data Availability

Not applicable.
